# Systematic review of the effectiveness of arts therapy for children and adolescents with post-traumatic stress disorder

**DOI:** 10.3389/fpsyt.2025.1716481

**Published:** 2025-12-11

**Authors:** Zehui Li, Qiaoyu Cui, Xinyu Liu

**Affiliations:** Department of Music Artificial Intelligence and Music Information Technology, Central Conservatory of Music, Beijing, China

**Keywords:** children, adolescents, arts therapy, systematic review, post-traumatic stress disorder

## Abstract

**Background:**

Arts therapy has gained increasing popularity for alleviating post-traumatic stress disorder (PTSD) in adolescents and children due to its non-intrusive nature and ease of interaction with participants. In this pre-registered systematic review (PROSPERO: 420251060744), we synthesized existing literature on arts therapies as interventions for managing PTSD symptoms in adolescents and children.

**Methods:**

We conducted systematic searches of APA PsycNET, PubMed, EMBASE, Cochrane Library, and Web of Science up to June 15^th^ 2025.

**Results:**

1,273 articles were identified through systematic searches, with 10 articles meeting our inclusion criteria. These studies reported some positive outcomes from visual art therapy, music therapy, dance therapy, drama therapy, and poetry therapy; however, the effectiveness of individual arts therapies in improving PTSD symptoms was inconclusive.

**Conclusion:**

Arts therapies demonstrate significant effects on certain symptoms of children and adolescents with PTSD. However, more rigorous studies are warranted to substantiate the efficacy and generalizability of arts therapies.

**Systematic review registration:**

https://www.crd.york.ac.uk/prospero/, identifier CRD420251060744.

## 1 Introduction

Post-traumatic stress disorder (PTSD) is a psychological disorder triggered by life-threatening or severe traumatic events (e.g., violence, abuse, natural disasters, war). Its core symptoms cover intrusive memories, avoidance behaviors, hypervigilance, and negative emotional/cognitive changes (1). Globally, children and adolescents are high-risk groups for PTSD. According to data from the National Center for PTSD (2025) in the United States (2), about 14% to 43% of children and adolescents will experience at least one traumatic event in their lifetime. PTSD impairs emotional regulation by triggering comorbid conditions such as depression and anxiety, disrupts attention, memory, and executive function to compromise academic performance, and leads to social withdrawal, creating a vicious cycle. It is accompanied by neurostructural abnormalities, including enlarged amygdala and delayed prefrontal cortex development, exerting far-reaching effects on the psychological, cognitive, and social functioning of children and adolescents (3–5).

Currently, interventions for childhood and adolescent PTSD primarily rely on cognitive behavioral therapy (CBT), particularly trauma-focused CBT (TF-CBT), which alleviates symptoms through cognitive restructuring, exposure therapy, and family involvement (6). Eye movement desensitization and reprocessing (EMDR) has also been validated as effective for childhood PTSD, with efficacy slightly superior to established trauma treatments (7). Although these methods have demonstrated efficacy in high-income Western countries, their application faces significant limitations when extended to resource-poor regions or culturally diverse populations. First, language and cognitive demands restrict applicability. TF-CBT relies on the ability to verbally describe traumatic events, yet children, particularly younger ones or those with impaired language function post-trauma, often struggle to accurately recount traumatic experiences, leading to treatment resistance (8). Second, insufficient cultural adaptability. EMDR’s bilateral stimulation techniques (such as eye movements) may be perceived as unusual or offensive in certain non-Western cultures, potentially triggering defensive responses in children (9). Therefore, novel therapeutic approaches that are safe, non-invasive, and readily interactive with children and adolescents need to be explored and validated.

Arts therapy utilizes nonverbal creative activities, like painting, music, and drama, to help individuals express traumatic experiences that are difficult to articulate verbally. It is considered a potential alternative or complementary intervention for childhood PTSD (10). Its core mechanism lies in: artistic creation transforms traumatic memories into concrete symbols through a symbolization process (e.g., shattered painting representing family breakdown), bypassing linguistic constraints to directly activate the nonverbal processing areas of the right brain, thereby reducing excessive arousal in the amygdala (11); simultaneously, group art activities rebuild social connections through peer interaction, counteracting the isolation caused by PTSD (12). Common types of arts therapy include visual art therapy, such as painting, collage, and sculpture (13); music therapy, encompassing improvisation, songwriting, or narrative musical expression (14); drama therapy, where children reconstruct traumatic narratives within a safe “fictional space” through role-playing (e.g., playing another self during the traumatic event) or improvised scenarios, thereby reducing perceived threats to their real identity (15). Dance/movement therapy facilitates the release of somatic traumatic memories (such as muscle tension and tremors) through bodily movements (e.g., free dance, rhythmic imitation), promoting the integration of mind and body (16).

The cross-diagnostic validity of arts therapy has been validated in multiple studies. Monti et al. (17) confirmed its effectiveness in improving psychosocial function among cancer patients; Corbett et al. (18) found that drama therapy can improve social deficits in adolescents with autism within a short period; Malhotra’s team (19) proposed that arts therapy may improve PTSD-related brain network dysfunctions through a triple network model (central executive network, default mode network, salience/emotion network). These lines of evidence provide direct support for arts therapy as an intervention tool for PTSD, particularly in scenarios where verbal expression is limited or cultural sensitivity is paramount. Early research has preliminarily validated the efficacy of art therapy. Pifalo’s (2002) (20) randomized controlled trial (RCT) demonstrated that children receiving 12 sessions of visual art therapy experienced a 42% reduction in core PTSD symptoms and a 63% decrease in social withdrawal, yielding significant outcomes. These findings provide preliminary evidence for the feasibility of art therapy for PTSD. However, Feen-Calligan (2020) (21) found that while visual arts therapy significantly improved separation anxiety symptoms, it showed no significant difference from the control group in overall PTSD symptom scores. Hylton et al. (2019) (22) also found in their pilot study that while arts therapy (including visual arts, drama, and music) significantly improved adolescents’ PTSD symptoms and depressive mood (p=0.03), the music therapy-only group did not achieve statistical significance in improving PTSD symptoms (p=0.09), anxiety (p=0.73), or depression (p=0.4). Due to the limited number of intervention studies on specific art therapies and the high heterogeneity across studies, meta-analysis is not feasible. Therefore, this research provides only a systematic review of relevant studies.

## 2 Methods

This was a pre-registered systematic review (PROSPERO registration number: CRD420251060744) conducted within the PRISMA framework. The review aimed to synthesize evidence on the therapeutic and health-promoting effects of arts therapy for mental health issues in children and adolescents with PTSD.

### 2.1 Inclusion criteria

Studies meeting the following criteria were included: (1) Study population: Participants were children (≤18 years) and adolescents (10–19 years) diagnosed with PTSD according to the WHO ICD-11 criteria; 2) Intervention measures: The intervention measures in the study must incorporate one or more forms of art therapy, such as painting, sketching, collage, puppet-making, sculpture, singing, listening to music, games, dance, drama, stage performance, storytelling, and lyric composition. (3) Study design: All types of study designs were accepted, including qualitative research, quantitative research, and mixed-methods research. Specific types encompassed intervention evaluations, pilot studies, quasi-experimental studies, pre-post studies, randomized controlled trials (RCTs), and non-RCTs. Studies may include a control group or be without a control group. (4) Outcome measures: Studies must report clear outcome measures, primarily focusing on psychological and physiological health symptoms, such as PTSD and anxiety. Core outcome measures included changes in total PTSD symptom scores assessed using standardized scales (e.g., ITQ, UCLA, CROPS, C-PTSD-C, CRTES); changes in anxiety symptom scores assessed using standardized scales (e.g., GAD-7, SCARED); reductions in depression and negative emotion scores, or increases in positive emotion scores.

### 2.2 Exclusion criteria

In this systematic review, we excluded the following types of literature: (1) studies where arts therapy was used in combination with other non-arts therapies (such as psychological or psychiatric therapies) or administered following other interventions; (2) studies involving adults (≥20 years old) or individuals not explicitly diagnosed with PTSD, or those with severe physical or mental comorbidities, such as autism, mental disorders, or cancer; (3) Studies classified as reviews, meta-analyses, editorials, conference abstracts, or case reports lacking clearly defined outcome measures; (4) Studies failing to report quantitative outcome measures related to mental health conditions, such as PTSD or anxiety.

### 2.3 Search strategy & search plan concept

A systematic search was conducted for English-language literature published up to June 15^th^ 2025. The included databases comprised APA Psychological Services Network (PsycNET), PubMed, EMBASE, Cochrane Library, and Web of Science.

The following search strategy was applied iteratively: Delayed Onset Post Traumatic Stress Disorder or Moral Injur* OR Post Traumatic Stress Disorder* OR Posttraumatic Neuroses OR Post-Traumatic Neuroses OR posttraumatic neurosis OR posttraumatic psychic syndrome OR posttraumatic psychosis OR posttraumatic stress OR post-traumatic stress OR Posttraumatic Stress Disorder OR Post-Traumatic Stress Disorder* OR Posttraumatic Stress Disorders OR posttraumatic syndrome OR PTSD OR trauma and stressor related disorders OR traumatic stress OR traumatic stress disorder* AND children OR child OR Adolescence OR adolescent OR Teen* OR Teenager* OR Youth* AND music therapy OR active music therapy OR active group music therapy OR expressive music therapy OR art therapy* OR art treatment OR sensory therapies OR dance therapy* OR dance movement psychotherapy OR dance movement therapy OR dance psychotherapy OR drama therapy OR dramatherapy OR drama psychotherapy OR poetry therapy.

### 2.4 Data extraction

Two researchers independently screened studies based on titles and abstracts. Any discrepancies between the first and second researchers were settled through discussion with a third researcher. Similarly, full-text reviews were conducted independently by two researchers, with disputes solved through consultation with the third researcher. Data extraction was performed using an Excel spreadsheet tailored to review objectives. This sheet contained predefined variables including author(s), publication year, country, study type, research method, interventions, setting and environment of implementation, sample size, age range, treatment duration, follow-up period, patient etiology, outcome measurement methods, and findings.

### 2.5 Quality and bias assessment

The methodological quality of RCTs was assessed using the Cochrane Risk of Bias tool version 2 (RoB 2) across five domains: randomization process, deviation from the intended intervention, missing outcome data, outcome measurement, and outcome reporting selection. The risk of bias was rated as “some concern”, “low”, and “some concern”, respectively, for these five domains. An overall bias assessment was derived for each outcome, with the overall risk of bias for each article determined by the highest risk category assigned in any domain. Two reviewers independently assessed study quality. Disagreements were settled through discussion; unresolved disagreements were resolved by a third co-author.

## 3 Results

### 3.1 Screening results

**Figure 1:**
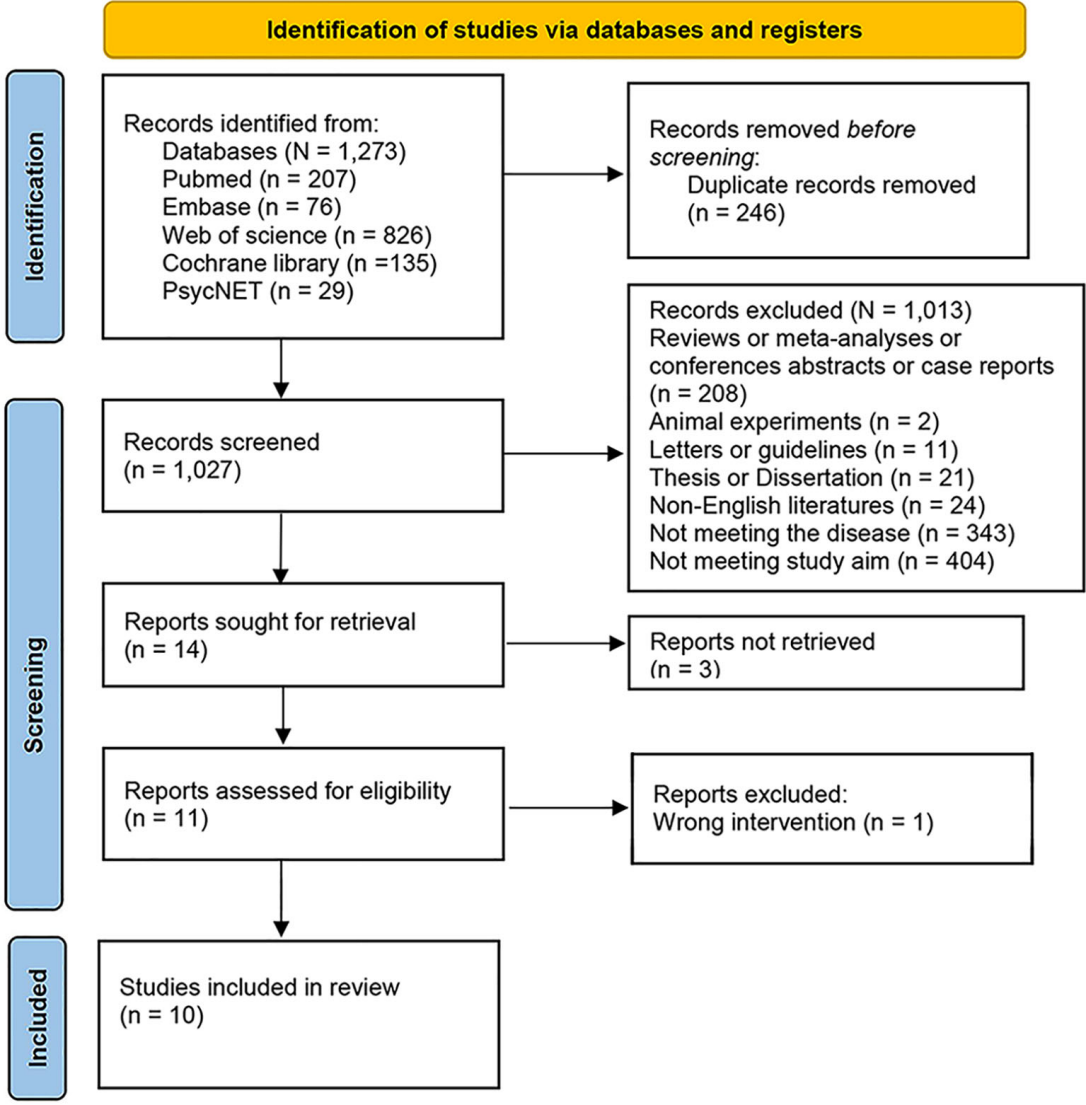
PRISMA chart.

This search yielded 1,273 articles, of which 246 were duplicates. 1,027 articles were screened for titles and abstracts, and 14 articles were selected for full-text review. Ultimately, 10 articles (21–30) were included in this review. The screening process and reasons for exclusion are reported in the PRISMA diagram shown in **Figure 1**.

### 3.2 Risk of bias

**Figure 2:**
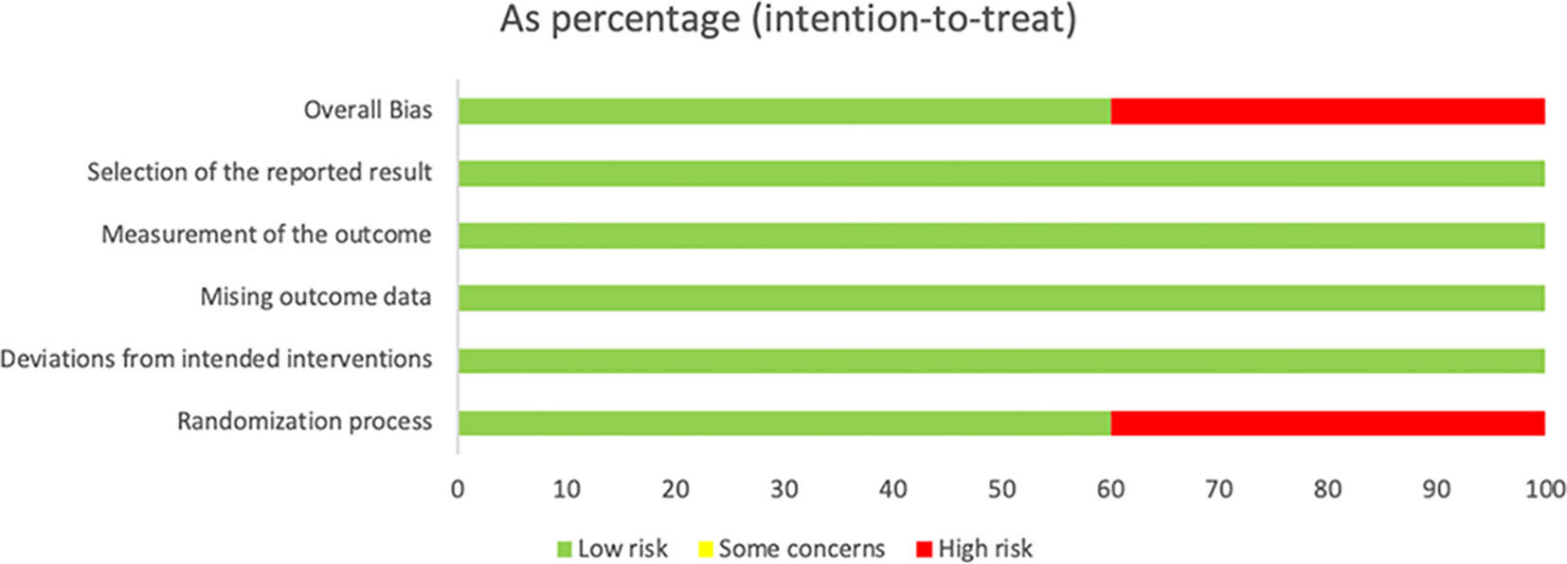
Risk of bias graph.

**Figure 3:**
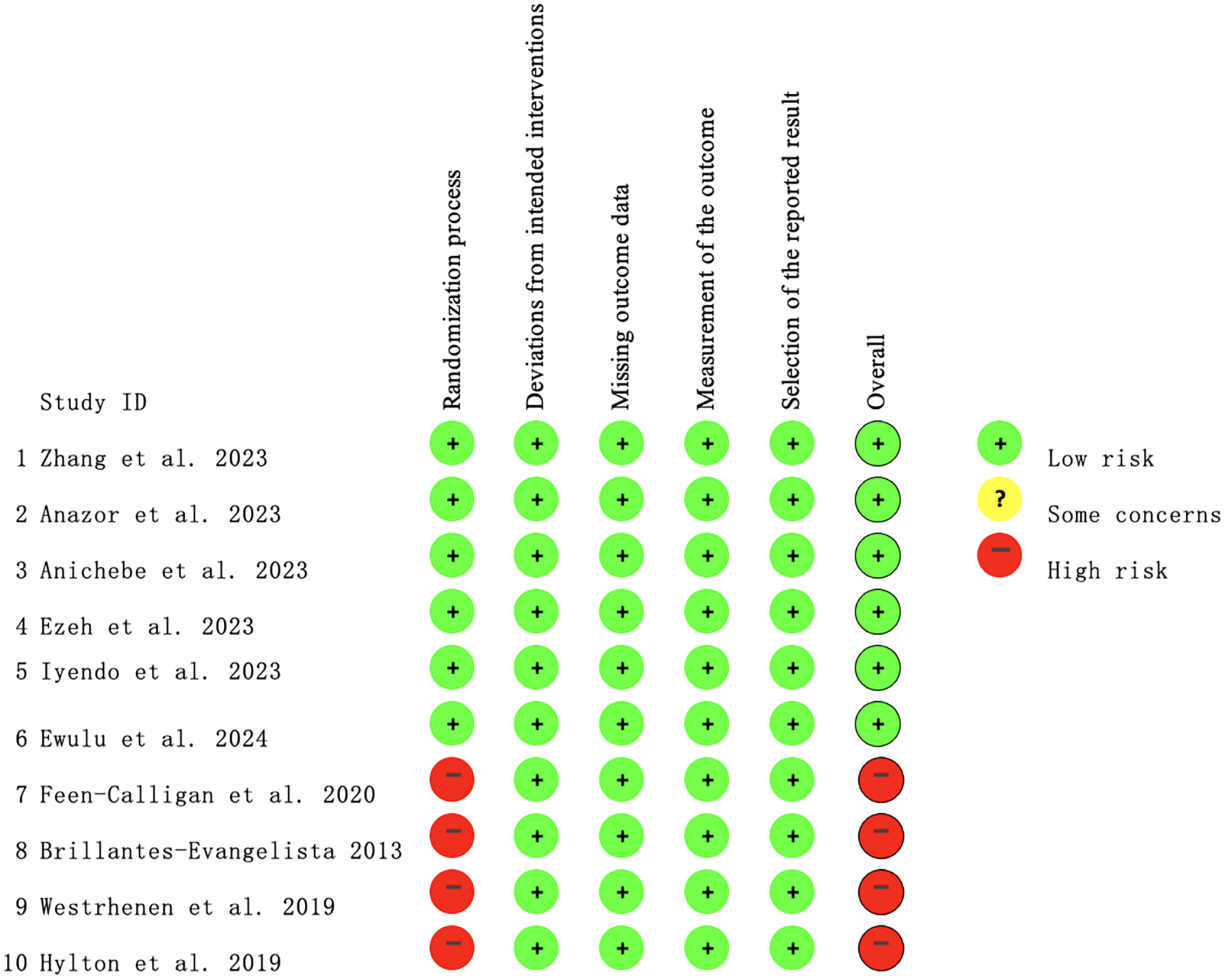
Risk of bias summary.

Overall quality assessments are displayed in **Figures 2** and **3**. Among the 10 studies, 2 were quasi-experimental studies, 1 was a non-RCT, and 1 was an observational study that did not employ randomization or allocation concealment, indicating a high risk of bias. The remaining studies were at low risk of bias.

### 3.3 Study characteristics

**Tables 1**, **2** provides an overview of the 10 included studies. These studies collectively enrolled 1,336 refugee adolescents, with sample sizes ranging from 11 to 200 participants. All studies were published between 2013 and 2024. Six studies were conducted in Nigeria, two in the United States, and the remaining studies were carried out in the Philippines and South Africa, respectively. All ten studies employing art therapy as a treatment modality were quantitative in design (including two RCTs, one non-RCT, six quasi-experimental studies, and one pilot evaluation report). Regarding the research settings, five studies were conducted in schools, one in a hospital, one at a summer camp, one at a community-based recreation center, and two did not specify their settings. Only one of these ten studies analyzed the effects before and after arts therapy; the remaining studies compared arts therapy with control groups, or even compared two or more arts therapy modalities across groups, including visual art therapy, dance therapy, music therapy, drama therapy, and poetry therapy. Five studies involved patients whose PTSD stemmed from kidnapping, two from abuse, one from flooding, one from a shooting incident, and one from a refugee crisis.

**Table 1:** Baseline characteristics.

First author	Year	Country	Study type	Study setting	Intervention	Sample size	Age range	Duration	Follow-up	Etiology	Details	Outcomes
Zhang (30)	2023	Northern region of Nigeria	RCT	School	Visual Art Therapy	78	/	6 weeks	3 months	Abduction	Sculpting, painting, and sketching	International Trauma Questionnaire (ITQ)
					Music Therapy	78	/				Listening to music, singing, playing instruments	
					Poetry Therapy	79	/				Reading existing suitable literature, writing poems and writing down their stories	
					Control Group	235	/					
											Routine Care	
Anazor (23)	2023	Nigeria	Quasi-experiment	School	Arts therapy	200	16-18 years	12 weeks	6 months	Abduction	Visual arts, dance, music, creative art	International Trauma Questionnaire (ITQ)
					Control Group	200						
											No Intervention	
Anichebe (24)	2024	Nigeria	Quasi-experiment	School	Drama Therapy	120	10-16 years	6 weeks	/	Flood	Watching the recorded drama between 55min and 1 h.	International Trauma Questionnaire (ITQ); GAD-7 scale
					Music therapy	120					Music storytelling	
					Control Group	122					No Intervention	
Ezeh (27)	2023	Nigeria	RCT	School	Visual Art Therapy	117	10-14 years	10 weeks	6 months	Abduction	Sketching	International Trauma Questionnaire (ITQ)
					Dance Therapy	117					Breathing exercises, performing dance step	
					Control Group	236					No Intervention	
Iyendo (28)	2023	Nigeria	Quasi-experiment	School	Music Therapy	117	10-18 years	6 weeks	3 months	Abduction	Listening to music, singing, playing instruments	International Trauma Questionnaire (ITQ)
					Visual Art Therapy	118					Drawing shapes, sketching down their feelings, drawing on canvas	
					Control Group	235						
											No Intervention	
Ewulu (26)	2024	Nigeria	Quasi-experiment	/	Music Therapy	49	10-16 years	10 weeks	4 months	Abduction	Listening to recorded music, watching music videos, discussing the music watched and listened to and asking the participants to mimic the music they have watched and listened to	International Trauma Questionnaire (ITQ); Family Happiness Scale
					Visual Art Therapy	52					Drawing, painting, photography and coloring	
					Control Group	107						
											No Intervention	
Feen-Calligan (21)	2020	USA	Quasi-experiment	Community-based recreation center	Visual Art Therapy	12	7-14 years	12 weeks	/	Refugee	Telling stories through collage, puppetry, and other media	the UCLA Child/Adolescent PTSD Reaction Index (UCLA); Screen for Child Anxiety Related Emotional Disorders (SCARED)
					Control Group	12						
											No Intervention	
Brillantes-Evangelista (25)	2013	Philippines	Quasi-experiment	Hospital	Poetry Therapy	11	/	8 weeks	/	Abuse	Tanaga, acrostic, word association, chronicles, couplet, free verse, letter-poem, community poem	Child Report on Post-traumatic Symptoms (CROPS); Self Rating Depression Scale
					Visual art therapy	11	/		/		Charcoal, Chinese brush and Indian ink, pastel colors, box, art papers, pens, memorable things, ceramic clay, pastel colors	
					Control Group	7						
											No Intervention	
Van Westrhenen (29)	2019	South Africa	Multicentre non-RCT	/	Creative Arts in Psychotherapy	23	7-13 years	10 weeks	/	Abuse	Visual art, movement, dance, drama, music, and storytelling	Child PTSD Checklist (C-PTSD-C); Child Behavior Checklist (CBCL) Posttraumatic Growth Inventory for Children- Revised (PTGI-C-R)
					Control Group	24						
											Court preparation and support program, no intervention	
Hylton (22)	2019	USA	Empirical research study	Summer camp	Creative Arts Group Therapy	34	9-12 grades	2 weeks	/	Shooting	Music: lyric analysis, life soundtrack, song writing/recording, group drumming;	Child’s Reaction to Traumatic Events Scale (CRTES); Positive and negative affect schedule (PANAS); Seven-Item Generalized Anxiety Disorder Scale (GAD-7); Patient health questionnaire-8 (PHQ-8); Satisfaction with the treatment program
											Visual Art: the mask, altered journal, graffiti mural, journey t-shirt;	
											Drama: role theory and method, projective technique, impro exercise, relationship lab,	

**Table 2:** Summary of findings from included studies.

First author	Intervention	Findings
Zhang (30)	Visual Art Therapy	Individuals receiving visual art therapy demonstrated a greater reduction in PTSD scores than those receiving music therapy or poetry therapy (p = 0.001); Three-month follow-up revealed that individuals receiving visual art therapy still exhibited a greater reduction in PTSD scores than those receiving music therapy or poetry therapy (p=0.001).
	Music Therapy	
	Poetry Therapy	
	Control Group	
Anazor (23)	Arts therapy	Interactive television arts therapy effectively alleviated PTSD symptoms in schoolchildren (p = 0.001); Follow-up at 6 months showed sustained improvement in symptoms among the treatment group.
	Control Group	
Anichebe (24)	Drama Therapy	Both drama therapy and music therapy significantly improved participants’ symptoms (p=0.001); Boys experienced more pronounced symptom reduction than girls (p=0.01)
	Music Therapy	
	Control Group	
Ezeh (27)	Visual Art Therapy	Participants in the visual art therapy and dance therapy groups showed decreased PTSD scores following the intervention (p = 0.03), with dance therapy yielding greater reductions than visual art therapy (p = 0.03); Follow-up assessments six months after visual art and dance therapy revealed sustained improvement in PTSD symptoms (p=0.001), with the dance therapy group demonstrating greater score reductions. This further indicates that dance therapy is more effective than visual art therapy.
	Dance Therapy	
	Control Group	
Iyendo (28)	Music Therapy	At the three-month follow-up, both visual art therapy and music therapy demonstrated sustained efficacy in alleviating PTSD symptoms (p = 0.001), with the visual art therapy group exhibiting a more pronounced reduction in PTSD scores, further indicating that visual art therapy is more effective than music therapy.
	Visual Art Therapy	
	Control Group	
Ewulu (26)	Music Therapy	Individuals receiving music therapy and visual art therapy demonstrated significant reductions in PTSD scores, with music therapy proving more effective in alleviating PTSD symptoms in children (p < 0.01)
	Visual Art Therapy	
	Control Group	
Feen-Calligan (21)	Visual Art Therapy	Compared with pre-intervention levels, participants receiving art therapy demonstrated significant improvement in separation anxiety symptoms (p < 0.01, Cohen’s d = 1.50). However, no significant changes were observed in PTSD symptoms, anxiety, panic disorder, generalized anxiety, school avoidance, or social anxiety scores. Compared with the untreated control group, individuals receiving art therapy demonstrated reduced PTSD symptoms (p = 0.05, Cohen’s d = 0.83)
	Control Group	
Brillantes-Evangelista (25)	Poetry Therapy	Participants receiving poetry therapy demonstrated a decrease in overall PTSD scores from pre-test to post-test. Scores rose slightly from pre-test to mid-test, but showed a significant decline from mid-test to post-test (p = 0.025). Participants who received visual art intervention demonstrated a significant decrease in PTSD scores in pre- and post-intervention assessments (p = 0.011).
	Visual Art Therapy	
	Control Group	
Van Westrhenen (29)	Creative Arts in Psychotherapy	Post-traumatic stress symptoms: The hyperarousal scores in the treatment group decreased significantly from a baseline mean of 10.39 to 6.77 (Cohen’s d = 0.61), with a mean difference of 4.36; Posttraumatic growth: The treatment group’s score increased from an average of 22.34 to 23.99 (Cohen’s d = 0.34), while the control group’s score rose from 19.75 to 23.44 (Cohen’s d = 0.72). However, there was no significant difference in the magnitude of increase between the two groups. Expressive art therapy effectively alleviated post-traumatic stress (p = 0.004, Cohen’s d = 0.54),
	Control Group	
Hylton (22)	Creative Arts Group Therapy	Expressive art therapy effectively alleviated post-traumatic stress (p = 0.004, Cohen’s d = 0.54), drama therapy significantly alleviated post-traumatic stress (p = 0.023), visual arts therapy increased positive emotions (p = 0.018) and reduced negative emotions (p = 0.003). Music therapy showed no significant effects.

### 3.4 Results of individual studies

#### 3.4.1 Visual art therapy

The outcome measures and results for each study are presented in **Tables 1**, **2**. Visual art therapy was the most frequently used intervention across these ten studies. Among them, Feen-Calligan (2020) (21) was the only study employing a single art therapy intervention. This was a 12-week art therapy program targeting Syrian refugee adolescents who had been living in the United States for about one year. PTSD symptoms and anxiety-related symptoms were assessed in 12 adolescents using the UCLA and SCARED scales. Following visual art therapy, participants demonstrated significantly reduced separation anxiety symptoms (p < 0.01, Cohen’s d = 1.50). Although no pronounced differences were observed in scores for PTSD symptoms, total anxiety symptoms, panic disorder symptoms, social anxiety, and generalized anxiety symptoms, improvements were noted (Cohen’s d ≥ 0.50). However, compared with the untreated control group, the visual art therapy group showed marked differences in changes for both PTSD and separation anxiety symptoms (p = 0.05), with large effect sizes (PTSD: Cohen’s d = 0.83; Separation anxiety: Cohen’s d = 0.84). For anxiety, panic disorder, and generalized anxiety, effects remained moderate (Cohen’s d > 0.50), while social anxiety showed a small effect (Cohen’s d = 0.41). Overall, arts therapy reduces PTSD-related symptoms, particularly separation anxiety and post-traumatic stress.

Additionally, several studies compared visual art therapy with other arts therapy modalities. Iyendo (2023) (28) and Ewulu (2024) (26) both employed interactive television-based art and music therapy in their interventions and compared the outcomes of the two approaches. Both studies utilized the ITQ to conduct pre- and post-tests on participants in the treatment and control groups. Iyendo et al. (2023) (28) revealed a notable main effect of interactive television art and music therapy on alleviating PTSD symptoms (p = 0.001), with visual art therapy proving more effective than music therapy (Art therapy group: M = 10.3, SD = 0.23; Music therapy group: M = 13.1, SD = 0.21; Tukey Honestly Significant Difference [HSD] test yielded p = 0.01). At the 3-month follow-up, both groups showed sustained improvement in PTSD symptoms, with the visual art therapy group exhibiting a greater reduction. Ewulu et al. (2024) (26) similarly found substantial differences in PTSD scores between music therapy and visual art therapy post-intervention (p = 0.001). However, they observed that music therapy was more effective in alleviating children’s PTSD symptoms (Art therapy group: M = 13.3, SD = 4.1; Music therapy group: M = 10.1, SD = 4.9).

Visual art therapy methods were also compared with other art therapy approaches. Brillantes-Evangelista et al. (2013) (25) compared the effectiveness of visual art and poetry interventions in improving PTSD symptoms and depressive symptoms. The visual art group showed a notable decrease in mean scores from pre-test to post-test (p = 0.011). Although the scores in the poetry group fluctuated during the mid-term, the overall scores decreased from pre-test to post-test (p = 0.025). The poetry group showed a notable decrease in mean pre-to-post-test scores on the self-rating depression scale (SDS) with a moderate effect size (p = 0.0445). However, no considerable differences were noticed in the mean pre-, mid-, and post-test scores between the visual arts and control groups. It is evident that visual arts and poetry contribute to alleviating PTSD symptoms, but only poetry therapy demonstrates significant efficacy in relieving depressive symptoms. Unlike Brillantes-Evangelista, Ezeh (2023) (27) compared visual art therapy with dance therapy. Results showed that participants in both visual art therapy and dance therapy groups exhibited decreased PTSD scores at post-intervention and 6-month follow-up assessments (p = 0.03). Tukey HSD revealed dance therapy to be more effective than visual art therapy in mitigating PTSD (p = 0.03). Follow-up assessments six months after both therapies indicated sustained improvement in PTSD symptoms (p = 0.1), with greater score reductions in the dance therapy group, further demonstrating dance therapy’s superior efficacy over visual art therapy.

Zhang (2023) (30) investigated the therapeutic effects of interactive television art, music, and poetry therapy on PTSD among Nigerian schoolchildren who experienced kidnapping. Results indicated that visual art therapy (M = 10.3, SD = 0.23) was more effective in relieving PTSD symptoms than music therapy (M = 13.1, SD = 0.21) and poetry therapy (M = 15.1, SD = 0.32) (p = 0.001). Similar results persisted at the three-month follow-up, further supporting the efficacy of arts therapy, particularly visual art therapy, in alleviating PTSD symptoms.

#### 3.4.2 Music therapy

Beyond the comparisons between visual art therapy and music therapy mentioned above (26, 28, 30), Obiora Anichebe et al. (2024) (24) also employed music therapy in their study. However, they focused on the effectiveness of drama therapy and music therapy in alleviating PTSD symptoms and generalized anxiety disorder (GAD) among schoolchildren affected by the 2022 floods in Nigeria. Overall, both drama therapy and music therapy demonstrated a combined main effect in relieving PTSD and GAD symptoms (p = 0.001), with a significant interaction effect for sex (p = 0.01). This indicated that boys reported greater symptom reduction than girls. Drama therapy was better in alleviating PTSD symptoms than music therapy, while music therapy was superior in alleviating GAD symptoms to drama therapy. However, statistical significance was not attained for these differences (p = 0.23).

#### 3.4.3 Arts therapy

Additionally, three studies examined arts therapy (also known as creative art therapy) as a therapeutic and counseling approach encompassing various art forms (visual arts, music, dance, and drama). These studies also employed controlled experiments to demonstrate the intervention’s effectiveness in alleviating PTSD symptoms. van Westrhenen (2019) (29) utilized four methods (visual arts, dance, drama, and music) in their research on the efficacy of creative art psychotherapy for traumatized children in South Africa. Results showed that excessive arousal symptom scores in the treatment group decreased significantly from a baseline mean of 10.39 to 6.77 at follow-up (Cohen’s d = 0.61); avoidance symptoms declined from 13.48 to 11.13 (d = 0.41), greatly more than in the control group (from 11.05 to 10.99, d = 0.01), with an adjusted mean difference of 4.11, though the effect size was small. Overall, PTSD symptoms and re-experiencing symptoms also alleviated in the treatment group, but no pronounced differences were recorded relative to the control group. Both the treatment and control groups exhibited a decline in behavioral problems over time. The score decreased from an average of 62.91 to 48.98 (d = 0.40) in the treatment group and from 71.35 to 51.46 (d = 0.61) in the control group. Internalizing behaviors decreased more than externalizing behaviors, but the difference was not statistically significant. Both the treatment and control groups showed increases in post-traumatic growth. It increased from an average of 22.34 to 23.99 (d = 0.34) in the treatment group and from 19.75 to 23.44 (d = 0.72) in the control group. However, there was no considerable difference in the magnitude of increase between the two groups. This study conducted creative art psychotherapy research in low-income settings in South Africa, offering new perspectives for treating PTSD children in the region. It found that creative art psychotherapy alleviated hyperarousal and avoidance symptoms, while its effects on other symptoms were not significant.

Anazor (2023) (23) also examined the therapeutic effects of interactive television art therapy (visual arts, dance art, music, and creative art) on PTSD symptoms among abducted schoolchildren in Nigeria through a quasi-experimental study. Results indicated a considerable main effect of arts therapy in mitigating PTSD symptoms (p = 0.001). At the 6-month follow-up, the average PTSD symptom scores in the treatment group continued to decline and remained at lower levels, whereas the control group maintained higher average scores. This suggested that interactive television-based art therapy effectively mitigated PTSD symptoms in schoolchildren, with sustained improvement observed in the treatment group at the 6-month follow-up.

Hylton et al. (2019) (22) conducted a pilot study in 2019 on creative art therapy for adolescent mental health following school shootings. The study involved adolescents who experienced the 2018 Parkland school shooting and included a 2-week creative art therapy camp (visual art, drama therapy, music therapy). They reveal significant increases in positive emotion scores post-treatment (p <.001, Cohen’s d = 0.81), a marked reduction in post-traumatic stress symptoms (p = 0.004, Cohen’s d = 0.54), alleviation of anxiety and depression symptoms (anxiety: p = 0.003, Cohen’s d = 0.52), and improvement of other negative emotions (p = 0.046, Cohen’s d = 0.42). They also conducted between-group comparisons of these three art therapies. Among them, drama therapy significantly reduced post-traumatic stress (p = 0.023), anxiety (p = 0.007), and depressive symptoms (p = 0.034) while markedly promoting positive emotions (p = 0.009). Visual art therapy increased positive emotions (p = 0.018) and reduced negative emotions (p = 0.003), but did not significantly reduce post-traumatic stress, depression, or anxiety symptoms. Music therapy did not significantly reduce post-traumatic stress, anxiety, depression, or negative emotions, nor did it significantly increase positive emotions. However, the music group exhibited the smallest sample size (n = 8), limiting its power to detect differences.

## 4 Discussion

This review delineated the application of arts therapy in alleviating symptoms among children and adolescents with PTSD. Based on the 10 included studies, arts therapy demonstrated effectiveness in improving PTSD symptoms. Furthermore, it showed efficacy in addressing PTSD-related generalized anxiety (22, 24), separation anxiety (21), depression (22, 25), positive emotions (22), and negative emotions (22).

Arts therapy has demonstrated significant effectiveness in improving PTSD symptoms. Additionally, previous studies indicate that other interventions also exert positive effects on PTSD. Among these, CBT and EMDR currently represent core evidence-based interventions for PTSD. Through cognitive restructuring (CBT) or bilateral sensory stimulation (EMDR), these approaches significantly reduce the intrusiveness of traumatic memories, avoidance behaviors, and hypervigilance symptoms (6, 7).The advantages of arts therapy may stem from its nonverbal expressive nature, offering a unique pathway to explore the pathological mechanisms of PTSD. First, it bypasses linguistic and cognitive defenses by utilizing nonverbal forms such as visual arts (painting, collage) and dance/movement (21, 27), enabling trauma survivors, particularly refugees and children with limited language skills, to naturally express emotions in a non-verbal pressure environment, thereby reducing psychological defense; Second, symbolization and integration: Through role-playing and symbolic creation (e.g., collages by Syrian refugees, dramatic narratives by flood-affected children) (22, 24), dramatic and visual arts transform fragmented traumatic memories into manageable symbolic expressions, facilitating the reconstruction of traumatic narratives and cognitive integration. Third, embodied regulation: Dance/movement therapy releases trauma-related muscular tension through bodily movement (27), while visual arts transform abstract emotions (e.g., fear, anger) into concrete pictures (e.g., colors/shapes in paintings), enabling embodied perception and regulation of emotions; Fourth, rebuilding social connections: Group-based arts therapy (drama, musical improvisation, community painting workshops) (24, 29) breaks the cycle of social isolation associated with PTSD through peer interaction and collaborative creation, restoring feelings of safety and belonging. These mechanisms intertwine, ultimately alleviating PTSD symptoms through non-threatening embodied participation, expression pathways resonating with cultural identity (23, 29), and neurobiological emotional regulation (e.g., theater reducing amygdala overactivation, visual arts activating prefrontal cognitive control) (22, 30).

Among the 10 studies included in this review, 7 explicitly stated that the intervention was conducted in a group therapy setting, while 3 did not specify the treatment setting. Within supportive groups, shared experiences and mutual support among members can foster a sense of belonging and reduce feelings of isolation. Furthermore, the sense of being accepted and nurtured gained through group interactions enables participants to experience inner healing and develop appropriate social interaction skills (21, 22, 25, 28, 29). Meanwhile, all interventions in the studies allowed children to engage in artistic creation rather than merely appreciating artworks, and each included sections for children to express their artistic ideas and share their thoughts through discussion. This approach not only helps children with PTSD detach from traumatic events and emotions while enhancing enjoyable artistic experiences (30) but also encourages them to fully express their understanding and feelings about traumatic events through various artistic modalities.

Visual art therapy has explored numerous forms, including sculpting, painting, sketching, photography, coloring, collage, and puppetry. Throughout this process, children are guided by art therapists to create beautiful pictures of nature or optimistic, positive patterns. They are also encouraged to freely express their thoughts and feelings through visual creation. Through tactile interaction with art materials and the support of therapeutic relationships, balanced arousal and focused attention are achieved, promoting physical and mental relaxation and a sense of security, thereby creating a safe psychological space for emotional processing (31). Artistic creation externalizes implicit experiences (e.g., bodily sensations) into visual symbols. Through reflection on the artwork, it facilitates the transformation of emotions from nonverbal bodily sensations to verbal expression, enhancing emotional awareness and integration capabilities (32). This approach helps enhance their problem-solving abilities, alleviate stress, engage in enjoyable activities, and improve emotional regulation and stability (23, 26–28, 30).

The core mechanism by which drama therapy facilitates psychological change through theatrical activities hinges on the dynamic equilibrium of aesthetic distance–enabling participants to safely explore, reenact, and rewrite traumatic narratives through role-playing, puppetry, or improvisation. This distance allows participants to engage with traumatic content but not to be completely overwhelmed by it, thereby enabling catharsis and cognitive restructuring within a protective framework. Psychologically, this facilitates a role transition from passive victim to active narrator (33, 34), while also enhancing empathy and social connections within group interactions, thus directly countering the isolation and disconnection often triggered by PTSD (24). The physical and verbal expressions employed in drama therapy are inherently similar to the experience of imaginative exposure (22), facilitating patients’ reassessment of traumatic experiences and alleviating associated PTSD symptoms. Moreover, drama therapy incorporates extensive physical movement through improvisation and story performance, which may contribute to emotional healing among trauma participants (35).

The classification of music therapy, drama therapy, and poetry therapy in intervention designs is largely consistent, primarily divided into two categories: presenting children with pre-selected artistic works (listening to recorded music, watching recorded films, and reading specific poems), and inviting children to participate in artistic creation (instrumental performance, musical storytelling, group drumming, role-playing, word association, and narrative activities) (23–26, 28, 30). The sense of rhythm in music and poetry recitation can enable emotional synchronization with participants. When analyzing lyrics and engaging in free poetic composition, participants project their emotions onto artistic works. Consequently, music therapy and poetry therapy hold potential positive effects in helping PTSD patients regulate emotions, release pent-up feelings, and integrate cognitive processes (25, 30). The distinction lies in music therapy’s greater reliance on nonverbal sense-reward pathways, making it suitable for populations with language expression difficulties or highly emotional traumatic memories (36). Poetry therapy, conversely, works through linguistic symbols–specifically narrative reconstruction (37). Both approaches regulate physiological arousal and social connection, yet their underlying neuropsychological pathways differ due to the distinct characteristics of their respective media.

Dance therapy engages participants in enjoyable activities through breathing exercises and dance movements, triggering emotional regulation, restoring a sense of hope, promoting relaxation, enhancing self-awareness, and teaching body scanning techniques. This may help reduce emotional distress and hyperarousal symptoms associated with PTSD (27, 29). Dance therapy promotes integration between the body and cerebral cortex by activating the right hemisphere (the region where traumatic memories are stored), alleviating trauma-related dissociation, and supplementing the limitations of traditional verbal therapy in addressing the limbic system (38).

The effectiveness of arts therapy in treating PTSD among children and adolescents suggests that visually oriented or dance-based interventions with low language barriers may be prioritized for patients with limited verbal expression, resistance to conventional therapies, or severe comorbidities. In resource-limited settings, simplified group art interventions could serve as first-line options but must be adapted to local cultural contexts (e.g., ritualistic dances, indigenous storytelling traditions). Additionally, therapists may provide personalized recommendations based on the child’s preferences, trauma type, and cultural background compatibility (e.g., kidnapping survivors may benefit from drama reconstruction to regain a sense of control; people with sexual trauma may be cautious about dance forms involving significant physical contact).

### 4.1 Practical implications

The findings of this study provide important clinical implications for schools, families, and healthcare institutions. For healthcare institutions, arts therapy can serve as a valuable supplement or alternative to traditional psychotherapy (such as CBT), particularly for children and adolescents with limited verbal expression or treatment resistance. Clinicians may prioritize recommending intervention methods with low language barriers, such as visual arts and dance, and tailor individualized treatment plans based on trauma type (e.g., using drama therapy for abduction survivors to rebuild a sense of control) and cultural background. At the school and community levels, simplified group art interventions should be promoted. These are not only cost-effective but also rebuild social connections through peer interaction, which can break the isolation experienced after trauma. Policymakers can support the introduction of such programs in resource-limited areas and encourage culturally adapted designs to enhance accessibility and cultural resonance. For families, children should be encouraged to participate in creative activities, such as painting and music, as safe channels for expressing emotions.

### 4.2 Strengths and limitations

This is a systematic review of arts therapy for adolescents and children with PTSD. Findings indicate that arts therapy, including visual art, music, dance, drama, and poetry, effectively alleviates symptoms in this population. The methodology involved comprehensive literature searches and analysis, with efforts to ensure comprehensiveness by incorporating grey literature.

However, our review also has limitations. First, the inclusion of both RCTs and non-RCTs may introduce bias from random assignment, necessitating cautious interpretation of results. Second, the intervention duration ranged from 2 to 12 weeks, with a wide span. While immediate post-intervention efficacy was observed across studies, only two studies included follow-up periods up to 6 months for long-term efficacy. This suggests potential long-term benefits, but further prospective RCTs are needed to confirm these effects. Third, the included studies were highly concentrated in a single cultural context (primarily Nigeria), severely limiting the generalizability of the findings to other cultural settings. Fourth, while the inclusion of gray literature ensures the comprehensiveness of the review, its lack of peer review and varying quality levels may introduce potential bias and uncertainty. Fifth, due to the team’s language limitations, only publicly available English-language literature was included, potentially overlooking significant research findings from non-English-speaking countries. Subsequent studies should establish multilingual teams or utilize professional translation tools to reduce bias arising from language screening. Sixth, self-reported scores in some studies may be subject to errors or subjective influence. Seventh, certain specific art therapy modalities, such as poetry therapy, have been studied in fewer studies, potentially introducing publication bias.

## 5 Conclusion

Arts therapy, encompassing visual arts, music, dance, drama, and poetry, shows potential efficacy in alleviating symptoms among adolescents and children with PTSD. However, due to the limited studies and the suboptimal quality of some included studies, we cannot comprehensively present the efficacy of various art therapy approaches in adolescents and children with PTSD, nor their long-term stability and universality. Nevertheless, arts therapy offers a flexible, scalable framework for managing PTSD in children and adolescents, demonstrating significant potential, particularly in resource-limited settings. Future research should isolate the independent effects of different arts therapies, extend follow-up periods, and optimize intervention effectiveness and universality through culturally adaptive designs. We recommend that clinicians consider incorporating arts therapy into their treatment plans for pediatric PTSD, and that policymakers support the implementation of arts therapy programs in schools, community centers, and mental health clinics.

## Data Availability

The original contributions presented in the study are included in the article/supplementary material. Further inquiries can be directed to the corresponding author.
